# Glutathionylation of beta-actin via a cysteinyl sulfenic acid intermediary

**DOI:** 10.1186/1471-2091-8-26

**Published:** 2007-12-10

**Authors:** Magnus Johansson, Mathias Lundberg

**Affiliations:** 1 Karolinska Institute, Department of Laboratory Medicine, Division for Clinical Microbiology, Karolinska University Hospital at Huddinge, S-141 86 Stockholm, Sweden

## Abstract

**Background:**

Cysteinyl residues in actin are glutathionylated, ie. form a mixed disulfide with glutathione, even in the absence of exogenous oxidative stress. Glutathionylation inhibits actin polymerization and reversible actin glutathionylation is a redox dependent mechanism for regulation of the cytoskeleton structure. The molecular mechanism that mediates actin glutathionylation *in vivo *is unclear.

**Results:**

We have studied glutathionylation of α- and β-actin *in vitro *using an enzyme-linked immunosorbant assay with a monoclonal anti-glutathione antibody. α- and β-actin were both glutathionylated when incubated with reduced glutathione (GSH) combined with diamide as a thiol oxidant. However, β-actin was also glutathionylated by both glutathione disulfide (GSSG) and GSH in the absence of diamide whereas α-actin was poorly glutathionylated by GSH or GSSG. Glutathionylation of β-actin by GSSG is likely to be mediated by a thiol-exchange mechanism whereas glutathionylation by GSH requires thiol oxidation. β-actin glutathionylation by GSH was inhibited by arsenite and dimedone suggesting that the mechanism involved formation of a cysteinyl sulfenic acid residue in β-actin.

**Conclusion:**

We conclude that glutathionylation of β-actin may occur via spontaneous oxidation of a cysteinyl residue to a sulfenic acid that readily reacts with GSH to form a mixed disulfide. We also show that the reactivity and oxidation to a reactive protein thiol intermediary differ between different actin isoforms.

## Background

Protein glutathionylation occurs by the formation of a mixed disulfide between a protein cysteinyl residue and glutathione [1,2]. It occurs in response to oxidative stress and has been suggested to be a mechanism to protect against irreversible oxidation of critical protein cysteinyl residues. Protein glutathionylation is reversible and reduction of the mixed glutathione-protein disulfides is efficiently catalyzed by glutaredoxins [3,4]. Several proteins have been identified by proteomic analysis as targets of glutathionylation in response to oxidative stress [5-7]. However, there is also evidence that protein glutathionylation may occur in the absence of exogenous oxidative stress and several studies suggest that it may be an important redox dependent signaling pathway and that glutathionylation directly regulates protein functions *in vivo *[1,2].

Actin was early identified as one of the most abundant protein that is glutathionylated in cells. Actin glutathionylation was first reported to occur in human neutrophiles stimulated with phorbol diesters to induce production of superoxide [8]. However, subsequent studies have shown that actin is constitutively glutathionylated in cells even in the absence of oxidative stress [9,10]. Glutathionylation efficiently inhibits actin polymerization and accordingly affects the cellular cytoskeleton structure [9-11]. Growth factors, such as epidermal growth factor, as well as interactions with the extracelllar matrix via integrin receptors has been shown to regulate actin polymerization by affecting the level of glutathionylation [9,12]. The molecular mechanism that mediates actin glutathionylation *in vivo *is unclear. Proposed mechanisms include oxidation of reduced glutathione (GSH) to glutathione disulfide (GSSG), which in turn can undergo thiol-disulfide exchange reactions with protein thiols to form glutathionyated proteins. However, under physiological conditions the concentration of GSH greatly exceeds the concentration of GSSG in cells, and unless the GSSG concentrations reach very high levels, GSSG unlikely glutathionylate proteins based on typical redox potentials of cysteinyl residues [13]. There is also several lines of experimental evidence against thiol-disulfide exchange with GSSG as the physiological mechanism mediating actin glutathionylation [14,15]. Other proposed mechanism includes formation of reactive glutathione species, such as glutathione-thiyl radicals, that can react with cysteinyl residues to form mixed disulfides.

Studies on actin glutathionylation have predominantly been performed on cell lines of non-muscle origin. However, several isoforms of actin exists in mammalian cells with differences in tissue distribution: α-actin is present in muscle cells whereas β- and γ-actin are components of the cytoskeleton in all non-muscle cells [16]. The actin isoforms show structural similarity with >90% identical primary structure. We have in the present paper studied glutathionylation of skeletal muscle α-actin and non-muscle β-actin *in vitro *using a highly sensitive enzyme-linked immunosorbant assay for detection of actin glutathionylation. In summary, we provide evidence that glutathionylation of β-actin occurs via spontaneous oxidation of a cysteinyl residue to a sulfenic acid that readily reacts with GSH to form a mixed disulfide.

## Results

### A highly sensitive ELISA for detection of actin glutathionylation *in vitro*

**Figure 1:**
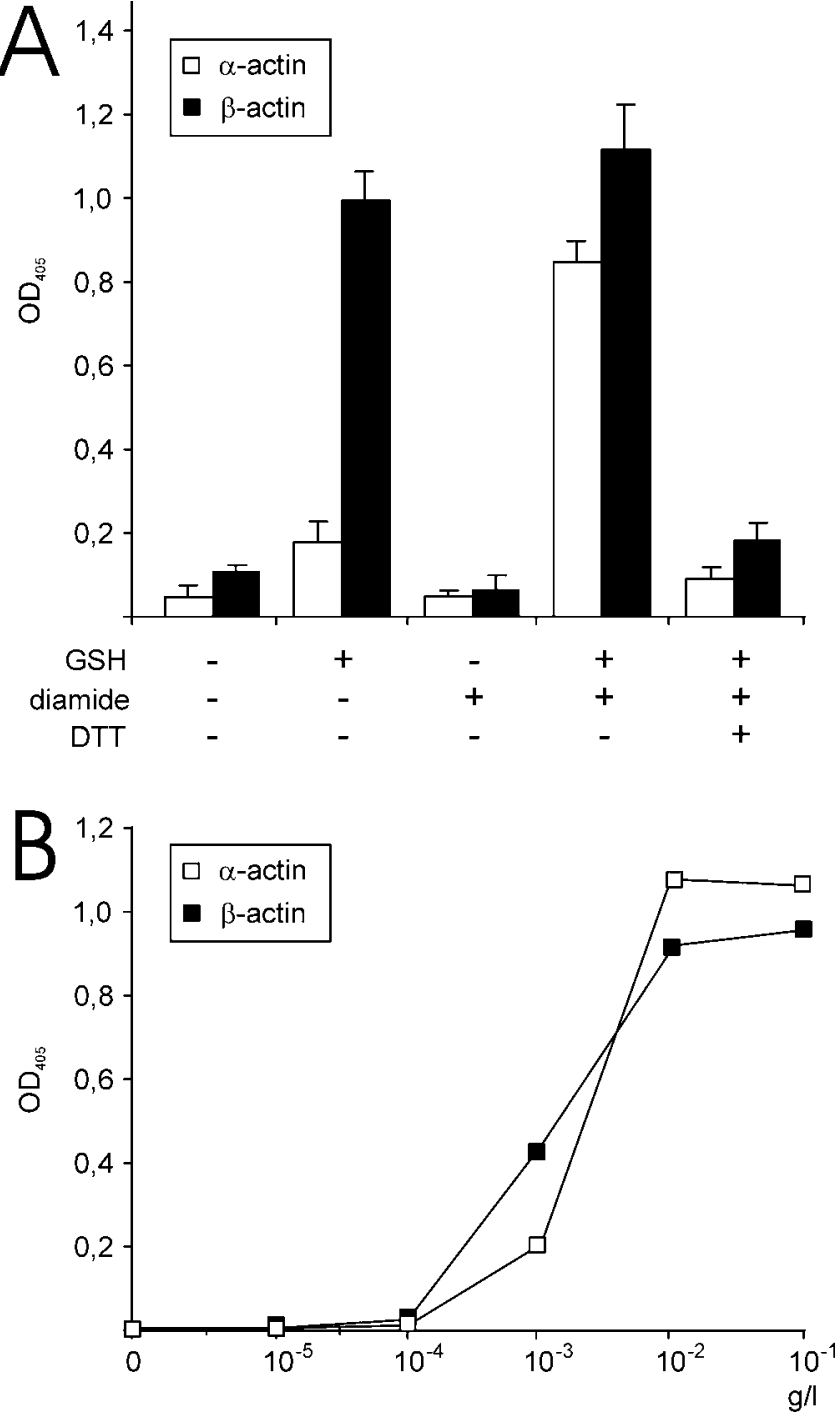
**Detection of actin glutathionylation using an anti-glutathione antibody ELISA**. (A) α- and β-actin coated on the plates were incubated with 1 mM GSH in the absence or presence of 1 mM diamide and 1 mM DTT. (B) Sensitivity of the assay for detection of glutathionylated actin coated on the plates.

We used a monoclonal anti-glutathione antibody to develop an ELISA for detection of actin glutathionylation *in vitro*. 96-well plates were coated with α- or β-actin and incubated with DTT to reduce any disulfides present in the samples. Diamide is a strong thiol-specific oxidant and incubation with diamide and GSH has successfully been used to glutathionylate actin *in vitro *[14,15]. We incubated the actins with combinations of 1 mM GSH and/or 1 mM diamide (Figure 1A). Actin glutathionylation was detected in the assay with an anti-glutathione antibody. No signal was detected in the wells that had not been coated with actin, independently from the incubation with DTT, GSH, diamide or any combinations of these reagents (data not shown). Accordingly, these reagents do not bind to the plates and interfere with the assay. A signal was detected when both α- and β-actin were incubated with GSH and diamide. The signal/background ratio for detection of the *in vitro *glutahionylated actin was ≈10:1. This signal was reduced to the background level when DTT was added to the samples. The decrease of the signal to background level by DTT reduction shows that the glutathione was bound to actin by a disulfide and not through non-covalent interactions. The plates were coated with different concentrations of actin (10^-5^–10^-1 ^g/l) to test the sensitivity of the assay (Figure 1B). The anti-glutathione antibody was used to detect glutathionylation after incubation with GSH and diamide as described above. Dilution of α- and β-actin to <10^-3 ^g/l during coating of the plates was sufficient for detection of actin glutathionylation. The coating of the proteins to the plate was performed in 50 μl/well, ie <50 ng (<1.2 pmol) actin per well was required for detection of glutathionylated actin in the assay. Accordingly, our data indicated that the assay was sensitive and specific for detection of actin glutathionylation *in vitro*.

An unexpected finding in the studies on actin glutathionylation was that β-actin, but not α-actin, was glutathionylated when incubated with GSH in the absence of diamide (Figure 1A). Similar to the diamide incubated samples, the ELISA signal decreased to background level when DTT was added to the samples indicating that the signal was due to the formation of a mixed disulfide between β-actin and glutathione (data not shown).

### β-actin is glutathionylated by GSH *in vitro*

**Figure 2:**
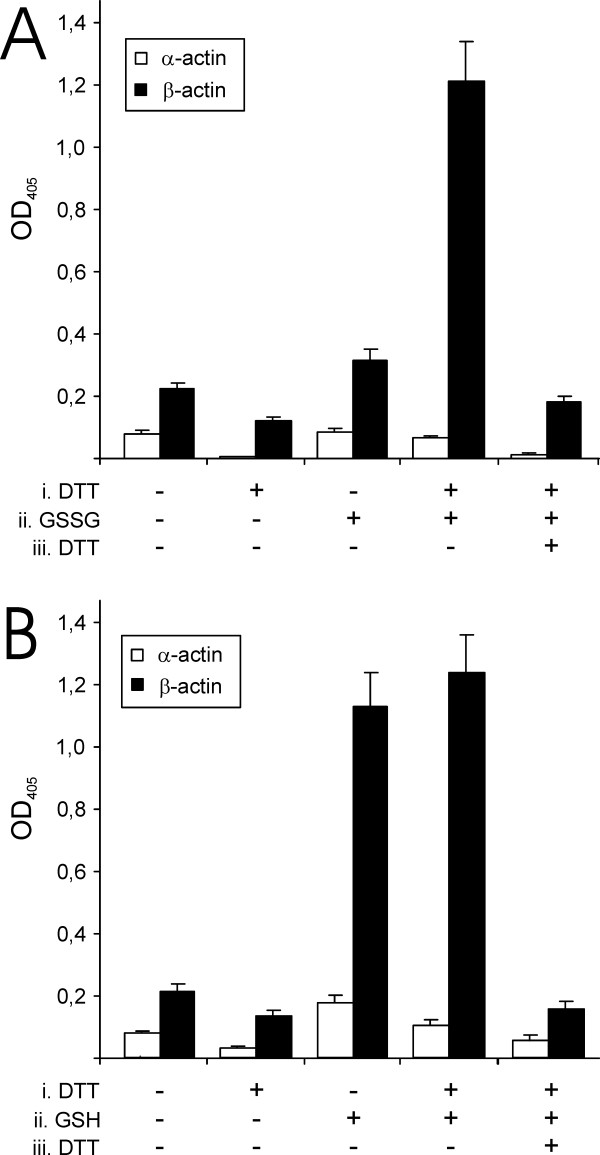
**Glutathionylation of α- and β-actin**. Glutathionylation of α- and β-actin by 1 mM GSSG (A) or 1 mM GSH (B). The indicated samples were incubated with (i) 1 mM DTT, after which 1 mM GSSG (A) or GSH (B) was added (ii) and a second incubation with 1 mM DTT was performed (iii).

We decided to further investigate and elucidate the mechanism of β-actin glutathionylation by GSH without diamide or other thiol oxidants present. α- or β-actin were sequentially treated as indicated in figures 2A and 2B with DTT to reduce any disulfides, incubated with 1 mM GSH or GSSG, and reduced again with DTT (Figure 2A and 2B). We did not detect any glutahionylation of α-actin above background levels when this actin isoform was incubated with either GSSG or GSH under any of these conditions. In contrast, β-actin was glutathionylated by both GSSG and GSH. Similar to the previous experiments, the ELISA signal for β-actin glutathionylation was decreased to background levels when DTT was added after glutathionylation. These findings suggested that β-actin was more reactive and easily glutathionylated by GSH or GSSG compared to α-actin.

**Figure 3:**
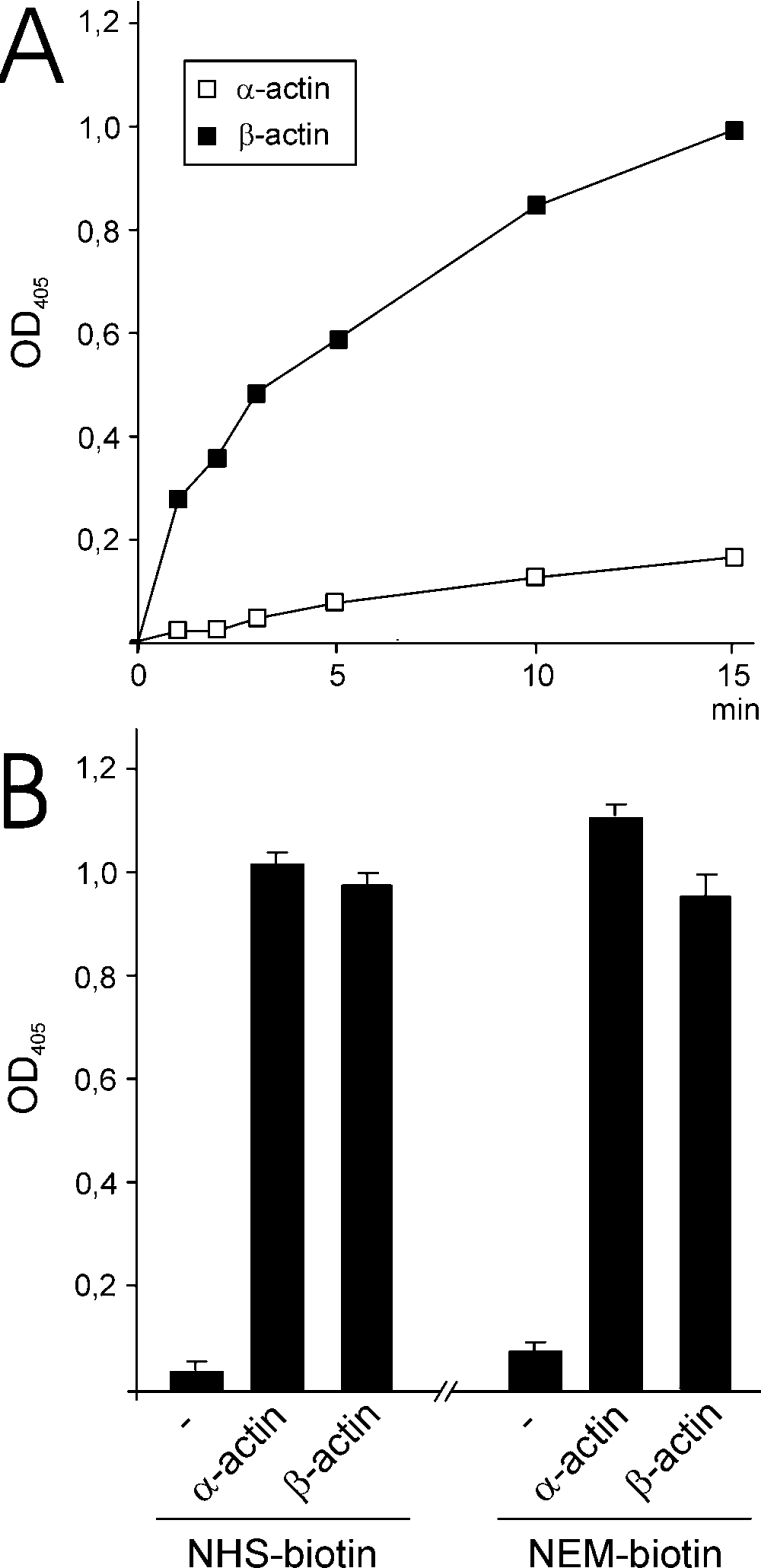
**Kinetics of α- and β-actin glutathionylation**. (A) Glutathionylation of α- and β-actin by GSH. The ELISA plates were coated with actin as described in Methods, incubated first with 5 mM DTT to reduce thiols and then incubated with 1 mM GSH at 22°C. (B) Quantification of relative amounts of actin immobilized on the plates (primary amine groups labelled with NHS-biotin) and accessible free thiols (thiols labelled with NEM-biotin).

The glutathionylation of β-actin by GSH was rapid and occurred within minutes of incubation whereas α-actin was poorly glutathionylated (Figure 3A). We decided to quantify the amounts of α- and β-actin immobilized on the plates to exclude that the differences in glutathionylation rate of was due to differences in amounts of the two actin isoforms coated on the ELISA plates. A highly selective biotinylation reagent (NHS-LC-biotin) was used to label primary amine groups of the α- and β-actin coated on the plates and we detected the biotinylated molecules with a streptavidin conjugate to determine the relative amount of proteins immobilized on the plates (Figure 3B). The amount of primary amines was similar for α- and β-actin, suggesting that the difference in glutathionylation was not due to difference in amount of proteins immobilized on the plates. We also used NEM-biotin to label free thiol groups to determine if there was a difference in accessible thiols between α- and β-actin. Similar to the labeling of the amine groups, no difference in accessible free thiols was observed for the proteins. Accordingly, these data suggest that the difference between glutathionylation of α- and β-actin was not due to difference in binding to the plates but rather a difference in the intrinsic reactivity of the proteins to GSH and GSSG.

**Figure 4:**
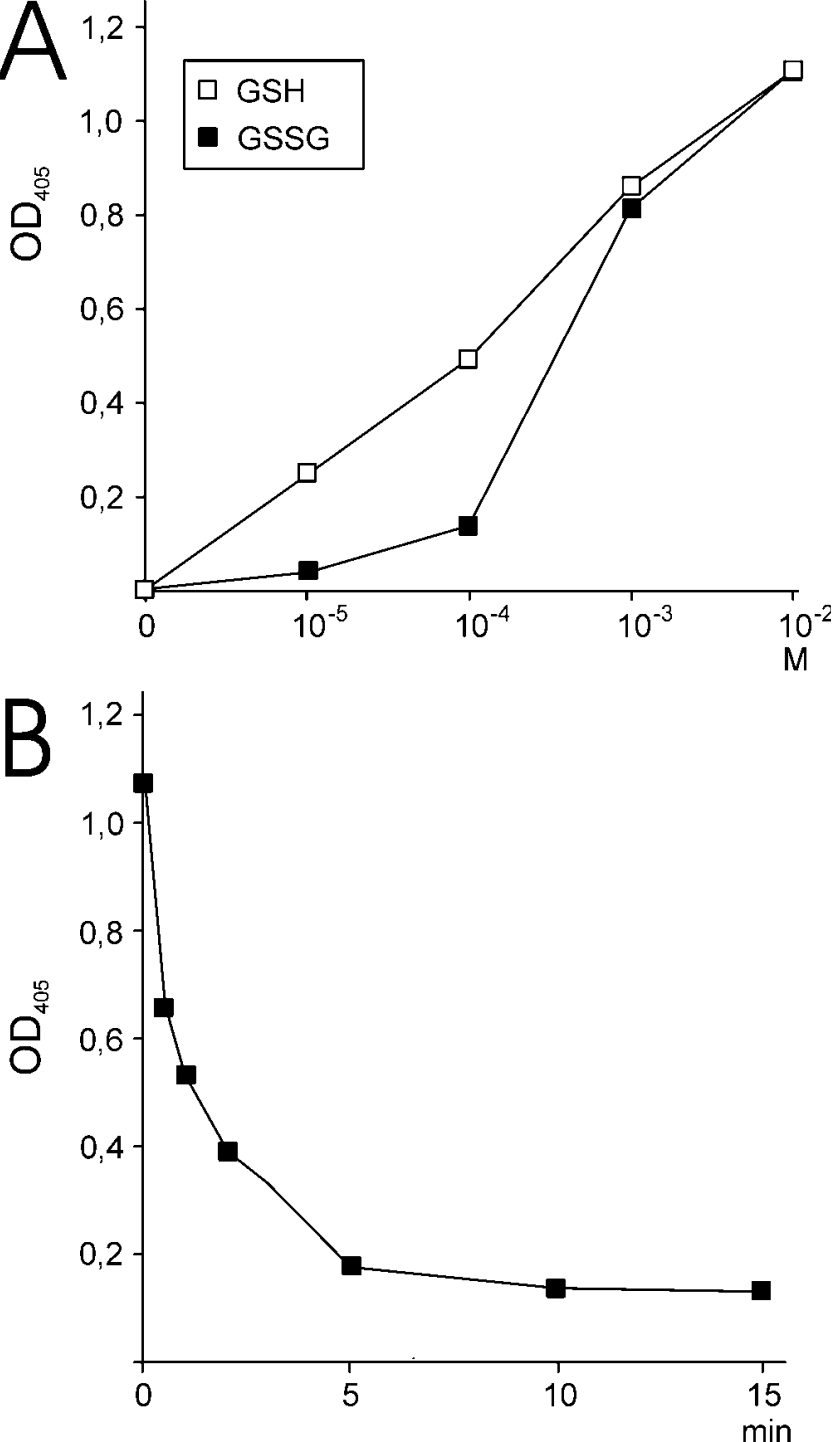
**Glutathionylation and deglutathionylation of β-actin**. Glutathionylation of actins by different concentrations of GSH and GSSG (A). Deglutathionylation of β-actin catalyzed by Grx1 (B)

Glutathionylation of β-actin by GSH requires a thiol oxidation. However, we could not exclude that the GSH used in the experiments had undergone partial oxidation to GSSG. If GSSG was present in the samples, one possible mechanism for β-actin glutathionylation was direct disulfide exchange between GSSG and actin thiols. However, we tested the concentrations of GSH and GSSG required to induce glutathionylation of β-actin (Figure 4A). These experiments showed that a lower concentration of GSH compared to GSSG resulted in glutathionylation of the protein. We also tested the effect of adding Grx1 to the reaction after 15 min incubation with GSH (Figure 4B). Addition of Grx1 resulted in reversal of the reaction and deglutathionylation of β-actin. These experiments excluded the possibility that glutathionylation of β-actin occurred by thiol-disulfide exchange with GSSG.

### Identification of a cysteinyl sulfenic acid intermediary in β-actin

**Figure 5:**
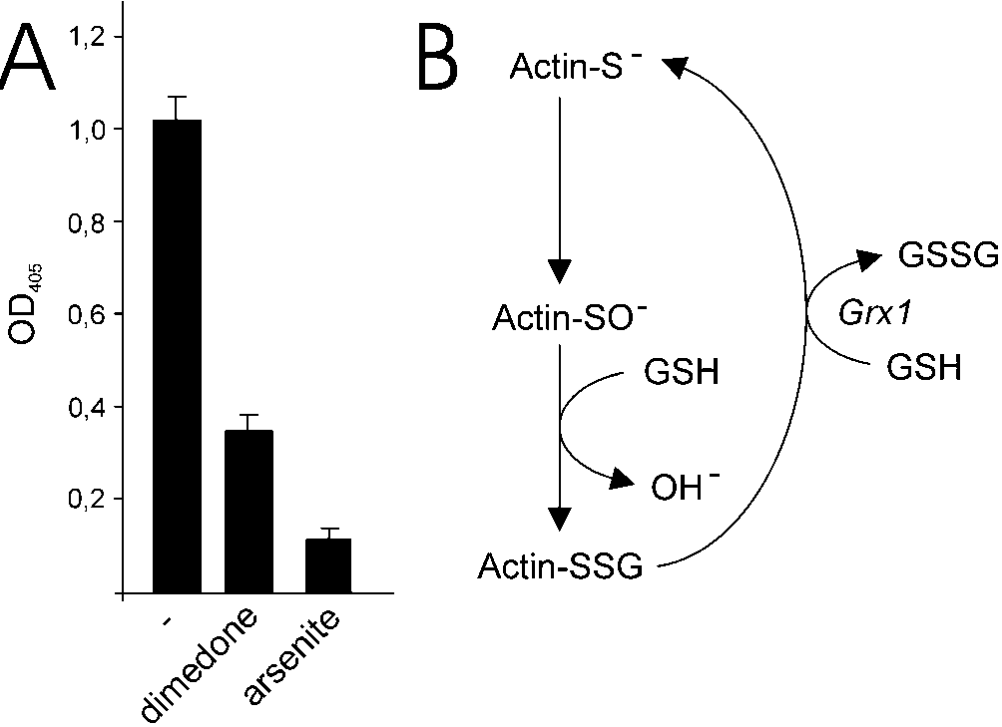
**Evidence of a cysteinyl sulfenic acid intermediary in β-actin**. Inhibition of β-actin glutathionylation by arsenite and dimedone (A). A model for glutathionylation of β-actin via a cysteinyl sulfenic acid intermediary (B).

Glutathionylation of actin by a mechanism independent of thiol-disulfide exchange suggested the formation of a reactive thiol intermediary. Oxidation of cysteinyl thiols to sulfenic acid derivatives have been identified in proteins such as the bacterial transcription factor OxyR and the redox regulated protein tyrosine phosphatase 1B (PTP1B) [17,18]. Cysteinyl sulfenic acids are susceptible to a nucleophilic attack by thiols and can readily react with GSH to form glutathionylated proteins [1]. Oxidation of the active-site cysteine in PTP1B to a sulfenic acid derivative occurs spontaneously in aerobic solution [19]. We hypothesized that a cysteinyl sulfenic acid intermediary was formed in human β-actin and that it reacted with GSH to glutathionylate the protein. Dimedone is a nucleophilic reagent that forms an adduct with sulfenic acids but does not react with reduced thiols [20,21]. Arsenite specifically reduces sulfenic acids to thiols but does not reduce disulfides [18,21]. Addition of either dimedone or arsenate to β-actin prior to addition of GSH resulted in a marked inhibition of β-actin glutationylation (Figure 5A). In summary, these data suggest that a cysteinyl residue in β-actin, but not α-actin, is spontaneously oxidized to a sulfenic acid that in turn rapidly reacts with GSH to glutathionylate the protein (Figure 5B).

## Discussion

Glutathionylation regulates actin polymerization and this is an important redox dependent mechanism of modulating cytoskeleton structure. Actin glutathionylation occurs even in the absence of exogenous oxidative stress and the mechanism mediating actin glutathionylation in the reducing intracellular environment is unclear as several studies provide evidence against direct thiol-exchange [14,15]. We have shown in the present study that β-actin is rapidly glutathionylated *in vitro *by GSH and we provide evidence that the reaction occurs via a cysteinyl sulfenic acid residue in β-actin that in turn reacts with GSH to form the protein-glutathione mixed disulfide. Oxidation of the actin cysteinyl to the corresponding sulfenic acid occurs readily without addition of an exogenous thiol oxidant, which suggests that the intrinsic properties of the actin cysteinyl render it highly susceptible to oxidization. Glutathionylation via a reactive cysteinyl intermediary explains how actin can be selectively glutathionylated in the reducing intracellular environment where the majority of protein thiols are reduced. It also explains how glutathionylation can occur even in the presence of high concentration of intracellular GSH as the reaction is independent on the relative concentrations of GSH and GSSG. Although our studies on actin glutathionylation have been performed *in vitro*, we find it likely that a similar reaction with a readily formed reactive thiol intermediary mediates actin glutathionylation *in vivo*.

We observed that glutathionylation of β-actin by GSH or GSSG was different if the protein had been treated with DTT or not before addition of glutathione (Figure 2). Incubation with GSSG only resulted in glutathionylation if the protein was reduced with DTT whereas incubation with GSH resulted in glutathionylation independent on DTT treatment. These findings are consistent with the formation of a cysteinyl sulfenic acid intermediary. DTT untreated β-actin has oxidized the target cystein to a cysteinyl sulfenic acid and is only glutathionylated by GSH. GSSG can not react with the sulfenic acid as there is no reducing moiety in the redox reaction. DTT treatment reduces the sulfenic acid to a thiol that can undergo thiol-exchange with GSSG to glutathionylate the protein. However, the thiol can also oxidize again to a sulfenic acid that in turn reacts with GSH to glutathionylate the protein.

Cysteinyl sulfenic acids have been identified in an increasing number of proteins in response to oxidative stress and exposure to thiol oxidants [21,22]. However, there is also evidence that cysteinyl sulfenic acids are formed in cells during constitutive metabolism without exposure to oxidative stress [21]. Oxidation of cysteinyl thiols by H_2_O_2 _is likely the major mechanism for formation for cysteinyl sulfenic acids under physiological conditions [21,22]. Low levels of H_2_O_2 _are produced endogenously and there is also evidence that H_2_O_2 _may be an important signaling molecule in cellular signal transduction pathways [23]. One example of such signaling pathway is induction of H_2_O_2 _production by epidermal growth factor that cause inactivation of PTP1B by reversible oxidation of an active site thiol in the enzyme [17,24]. PTP1B cystein 215 is oxidized to a sulfenic acid by H_2_O_2 _and this cysteinyl sulfenic acid may be further irreversibly oxidized to sulfinic or sulfonic acids. However, rapid reaction with GSH and glutathionylation of the active site cysteinyl sulfenic acid prevents oxidation to an irreversible oxidation state [25,26]. We hypothesize that actin glutathionylation may occur by a mechanism similar to PTP1B glutathionylation in response to H_2_O_2 _produced endogenously in cells. Glutathionylation of proteins via a sulfenic acid intermediary has been suggested to occur in other proteins as well and it may be a general mechanism in cells to mediate protein glutationylation [1,2,22,27].

Protein glutathionylation is reversible and the level of glutathionylated actin is determined by both the rate of glutathionylation as well as the rate of deglutathionylation. Our data suggest that actin glutathionylation occurs non-enzymatically via a cysteinyl intermediary whereas actin deglutathionylation occurs by thiol-exchange with GSH catalyzed by Grx1 (Figure 4B). The level of glutathionylated β-actin is, according to our model, determined by the Grx1 activity and the rate of formation of the cysteinyl sulfenic acid and the non-enzymatic reaction between the sulfenic acid and GSH. This is highlighted in the experiment presented in Figure 4B where β-actin was incubated with GSH to first glutathionylate the protein and Grx1 was subsequently added. Addition of Grx1, without addition of GSH or another reducing agent, resulted in rapid reversal of the reaction and deglutathionylation the protein. This experiment supports that protein glutathionylation and deglutathionylation can occur in parallel in a GSH containing redox buffer. This model of actin glutathionylation explains how actin can become glutathionylated even in the presence of high concentrations of GSH as is present intracellularly. GSH is inefficient by itself to reduce the glutathionylated actin but addition of Grx1 rapidly reduces the mixed disulfide via thiol-exchange with GSH.

α- and β-actin have >90% conserved primary structure but the number of cysteinyl residues differ between the actin isoforms [16]. Muscle α-actin has five cysteinyl residues whereas non-muscle β- and γ-actin have six cysteinyl residues. The C-terminal located cystein 374, that is conserved in all actin isoforms, has been identified as a target for glutathionylation in α-actin [9,11]. However, a recent study by Lassing and co-workers identified cystein 272 as the most reactive cysteinyl residue in β-actin [28]. This cysteinyl residue is unique to β- and γ-actin as α-actin does not contain a cysteinyl residue in the corresponding structural location. Cystein 272 is located at the protein surface and is easily oxidized by H_2_O_2 _[28]. We find it likely that the presence of cystein 272 in β-actin explains that the formation of a cysteinyl sulfenic acid only occurs in β-actin and not in α-actin. Our findings suggest that redox regulation of actin polymerization via reactive protein thiols and protein glutathionylation differ between skeletal muscle compared to other tissues where β-actin is a major component of the cytoskeleton.

## Conclusion

We provide evidence that β-actin glutathionylation may occur by a mechanism involving the spontaneous oxidation of a cysteinyl residue to a sulfenic acid. The reactive cysteinyl sulfenic acid can readily react with GSH to form a mixed disulfide and glutathionylate the protein. These finding explain how β-actin may be glutathionylated in the reducing environment of the cytosol.

## Methods

Human platelet β-actin and rabbit skeletal muscle α-actin were obtained from Cytoskeleton Inc. (Denver, CO, USA). 96-well Maxi-Sorp ELISA plates (Nunc Inc) were coated with 1 μg/ml α- or β-actin in pH 9.6 carbonate buffer for 30 min at 22°C. Thiols were reduced with 5 mM DTT in PBSET (phosphate buffered saline, pH 7.4 with 1 mM EDTA and 0.05% Tween-20) for 15 min at 37°C. The plates were washed four times with PBST (phosphate buffered saline, pH 7.4 with 0.05% Tween-20). GSH or GSSG were added with or without diamide (Sigma) as indicated and the samples incubated 15 min at 22°C unless other incubation times were stated. The deglutathionylation assay with human recombinant glutaredoxin 1 (Grx1) (IMCO Corporation Ltd AB, Stockholm, Sweden) was performed with β-actin coated and reduced on the plates as described above. 1 mM GSH was added to the plates incubated at 22°C. Grx1 (1 μM) was added in the same GSH buffer at 15 min and incubation continued to the time-points indicated. 5,5-dimethyl-1,3-cyclohexanedionedimedone (dimedone) (Sigma) or arsenite was added as indicated. All reactions was terminated by washing the wells four times with PBST.

Actin glutationylation was detected with a monoclonal anti-glutathione antibody (Research Diagnostics, MA, USA. Inc). The antibody was diluted 1:1000 in PBST supplemented with 1% (w/v) fraction V bovine serum albumin (Sigma) and added to the samples for 1 h incubation. The plates were washed four times in PBST and an anti-mouse alkaline phosphatase conjugated antibody (Sigma) diluted 1:1000 in PBST with 1% (w/v) fraction V bovine serum albumin was added and incubated for 1 h. The plates were washed four times in PBST and 1 mg/ml p-nitrophenyl-phosphate (Sigma) dissolved in 10% diethanolamine pH 9.8 with 0.5 mM MgCl_2 _was added. Absorbance at 405 nm was determined using a micro-titer plate reader (Wallac).

Relative amounts of α- or β-actin immobilized on the ELISA plates were determined by biotinylating primary amine groups of the proteins. The actins were coated on the plates and 2 μM Sulfo-NHS-LC-Biotin (Pierce Biotechnology Inc.) in PBST was added and incubated 15 min at 22°C. Free protein thiols were quantified after reduction with 5 mM DTT in PBSET by biotinylation of thiol groups. 2 μM of NEM-biotin (Sigma) was added in PBST and incubated as described above. The plates were washed with PBST and the biotinylated proteins detected by 30 min incubation with streptavidin-alkaline phosphatase (Mabtech AB, Stockholm, Sweden) diluted 1:1000 in PBST with 1% (w/v) fraction V bovine serum albumin. The plates were developed with p-nitrophenyl-phosphate as described above.

## Abbreviations

The abbreviations used are: Grx1, glutaredoxin 1; GSH, reduced glutathione; GSSG, glutathione disulfide

## Authors' contributions

The authors contributed equally to all parts of the project.
